# Pentraxin 3 promotes long-term cerebral blood flow recovery, angiogenesis, and neuronal survival after stroke

**DOI:** 10.1007/s00109-018-1698-6

**Published:** 2018-10-13

**Authors:** Ivana Rajkovic, Raymond Wong, Eloise Lemarchand, Jack Rivers-Auty, Olivera Rajkovic, Cecilia Garlanda, Stuart M. Allan, Emmanuel Pinteaux

**Affiliations:** 1 Faculty of Biology, Medicine and Health, AV Hill Building, The University of Manchester, Oxford Road, Manchester, M13 9PL UK; 2 Department of Immunology and Inflammation, Humanitas Clinical and Research Center, 20089 Rozzano, MI Italy

**Keywords:** Ischaemic stroke, Cerebral blood flow, Angiogenesis, Vascular remodelling, Neuroprotection

## Abstract

**Abstract:**

Restoration of cerebral blood flow (CBF) and upregulation of angiogenesis are crucial for brain repair and functional recovery after cerebral ischaemia. Pentraxin 3 (PTX3) is a key regulator of angiogenesis and is emerging as a promising target for cerebrovascular repair after stroke. Here, we investigated for the first time the role of PTX3 in long-term CBF, angiogenesis, and neuronal viability after ischaemic stroke induced by transient middle cerebral artery occlusion (MCAo). Lack of PTX3 had no effect on early brain damage, but significantly impaired restoration of CBF, 14 and 28 days after MCAo, compared to wild-type (WT) mice. Immunohistochemical analysis revealed that PTX3 KO mice have significantly greater neuronal loss, significantly decreased vessel diameter, vessel proliferation, vascular density, and reactive astrocytes and decreased expression of vascular endothelial growth factor receptor 2 (VEGR2), vascular extracellular matrix (ECM)-proteins (collagen IV, laminin), and integrin-β, in the ipsilateral (stroke) hemisphere compared to WT mice, 28 days after MCAo. Therefore, PTX3 promotes sustained long-term recovery of CBF, angiogenesis, and neuronal viability after cerebral ischaemia. Collectively, these findings demonstrate the potential and clinical relevance of PTX3 as a promising therapeutic target, providing sustained long-term post-stroke neurovascular repair and reducing the loss of neurons.

**Key messages:**

- Pentraxin 3 (PTX3) is a key regulator of angiogenesis and is emerging as a promising target for cerebrovascular repair after stroke.
- Restoration of cerebral blood flow (CBF) and angiogenesis are crucial for brain repair and functional recovery after cerebral ischaemia.
- PTX3 promotes sustained long-term recovery of CBF, angiogenesis, and neuronal viability after cerebral ischaemia.

**Electronic supplementary material:**

The online version of this article (10.1007/s00109-018-1698-6) contains supplementary material, which is available to authorized users.

## Introduction

A rapid and sustained re-establishment of normal cerebral blood flow (CBF) after ischaemic stroke (known as reperfusion) is associated with a better outcome and improved functional recovery. Indeed, clinical data suggests that ischaemic stroke patients that exhibit greater CBF show neurological improvements [1], whereas pre-clinical evidence indicates that a reduction in CBF post-stroke is associated with increased brain damage in rats [2]. Furthermore, experimental studies have reported that angiogenesis leads to the formation of new blood vessels after ischaemia and promotes recovery [3].

A new regulator of angiogenesis is the acute phase protein pentraxin 3 (PTX3), the function of which in cardiovascular and cerebrovascular disease is emerging [4, 5]. We have previously shown that brain PTX3 expression is upregulated after experimental stroke in the ipsilateral (stroke) hemisphere in mice, promoting recovery in the brain by reducing oedema and improving blood-brain barrier (BBB) integrity by enhancing glial scar formation [6]. Furthermore, previous studies have reported a reduction in the number of capillaries in PTX3 knockout (KO) mice in a model of acute myocardial infarction [4]. Published data from our group suggests that PTX3 promotes angiogenesis 14 days after experimental stroke, demonstrated by a decrease in vasculature, fewer newly formed blood vessels, and reduced expression of vascular endothelial growth factor receptor 2 (VEGFR2) in PTX3 KO mice [5]. Despite these findings, the effect of PTX3 on CBF is currently completely unknown, and the mechanisms underlying PTX3 regulation of post-stroke angiogenesis remain incompletely understood. In addition, whether pro-angiogenic actions of PTX3 are a transient or sustained long-term response is currently unknown.

Here, we show for the first time that PTX3 promotes sustained long-term recovery of CBF after experimental cerebral ischaemia. Crucially, we demonstrate a long-lasting pro-angiogenic effect of PTX3, revealed by a reduction in vessel diameter, proliferation of vessels, cerebrovasculature, and VEGFR2 expression, in PTX3 KO mice compared to wild-type (WT) mice, 28 days after stroke. PTX3 also upregulates vascular integrin-β1 expression in the stroke region of WT mice, but not in PTX3 KO mice. Moreover, integrin-β1 expression is significantly reduced compared to WT mice in the ipsilateral hemisphere. Interestingly, our study indicates that PTX3 promotes expression of both vascular and peri-vascular reactive (glial fibrillary acidic protein (GFAP) positive (+)) astrocytes in ischaemic regions, which was previously unknown. Finally, we show that PTX3 promotes long-term neuronal viability after experimental stroke. Collectively, our results suggest that PTX3 may be an effective post-stroke treatment option reducing neuronal loss and promoting long-term repair.

## Methods

### Animals

PTX3 KO mice and WT littermates were from heterozygote mice (obtained from Dr. Cecilia Garlanda, Humanitas Clinical and Research Center, Rozzano, Italy) and were genotyped as described previously [7]. All mice were bred on a C57BL/6 background. All animal procedures were carried out in accordance with the Animal Scientific Procedures Act (1986) and the European Council Directive 2010/63/EU and were approved by the Home Office and Animal Welfare and Ethics Review Board, University of Manchester (UK). All experiments adhered to the ARRIVE guidelines and IMPROVE guidelines [8, 9]. Surgical procedures and behavioural tests were carried out by an experimenter blinded to the genotype.

### Cerebral ischaemia induced by transient middle cerebral artery occlusion (MCAo)

Induction of anaesthesia was achieved by inhalation of 4% isoflurane (30% oxygen and 70% nitrous oxide gas, AbbVie Ltd., UK) and was maintained at 1.75% isoflurane. During surgery, a rectal probe was used to assess body temperature, which was kept at 37 °C ± 0.5 °C with a heat blanket (Harvard Apparatus, Edenbridge, Kent, UK). A laser Doppler monitor (Oxford Optronix, Abingdon, UK) was used to confirm a drop in CBF in the middle cerebral artery (MCA) region following occlusion and subsequent recovery of CBF. Focal cerebral ischaemia was achieved by MCAo using a protocol described previously as follows; prior to incision, topical anaesthetic (EMLA, 5% prilocaine and lidocaine, AstraZeneca, UK) was administered on to the areas of skin requiring incision. A laser-Doppler probe located 6-mm lateral and 2-mm posterior from bregma was fixed on to the skull with vetbond. An incision was made into the middle front section of the neck, and the right common carotid artery was exposed and ligated. The internal carotid artery received temporary ligation. An incision in to the common carotid artery was made, followed by the insertion and advancement of a 6–0 monofilament (Doccol, Sharon, MA, USA) through the internal carotid artery. After 15 min of occlusion (a short occlusion time was used to reduce mortality), the filament was removed allowing reperfusion. The neck wound was sutured, and saline (0.5 ml, for rehydration) and buprenorphine (0.05 mg/kg, providing general analgesia) were administered subcutaneously (sc). Animals were allowed to recover in an incubator (26 °C) and then held in ventilated cages placed on a heat pad (24 °C) under standard laboratory conditions with ad libitum access to mashed food and water for 24 h. Finally, cages were returned to their home rack and kept under normal housing conditions. All animals in our studies demonstrated a minimum of 70% decrease in CBF due to ischaemia. For the 28-day study, three WT and four PTX3 KO mice were culled at 24 h post-surgery due to ischaemic damage deemed too severe as per the IMPROVE guidelines. For the 48 h time-point study, one WT mouse was culled at 24 h due to severe ischaemic damage. Furthermore, one WT and one PTX3 KO mice from the laser speckle study were culled at 24 h post-surgery due to ill health [10].

### Laser speckle contrast imaging (LSCI) and analysis

Mice were anaesthetized with inhalation of 4% isoflurane and placed in a stereotaxic frame (World Precision Instruments, USA) on a heat mat set to 37 °C, positioned under a moorFLPI2 Full-Field Perfusion Imager (Moor instruments, UK). Anaesthesia was maintained at 1.75% isoflurane. The skin on top of the skull was opened to expose the skull bone, which was kept intact, and LSCI was conducted for 2 min at 100 frames (4 s per frame). Mice were imaged prior to MCAo (baseline recording), 72 h, 14 days, and 28 days post-MCAo. Two regions of interest (ROIs) located in the primary MCA area and frontal cortical regions were identified and used for analysis. To evaluate CBF, 15 images per mouse for each time point were analysed using moorFLPI2 Full-Field Laser Perfusion Imager Review V5.0 software. For all animals, flux values at each time point were measured for each ROI in the ipsilateral and corresponding ROIs in the contralateral hemisphere, and ipsilateral flux was expressed as a % of contralateral flux. Analysis was conducted under blinded conditions.

### Tissue processing

Animals were anaesthetised with 4% isoflurane and transcardially perfused with 0.9% saline followed by 4% paraformaldehyde (PFA) 48 h, 7 days, 14 days, or 28 days after MCAo surgery (see Supplemental materials for more detail).

### Infarct volume analysis

Cresyl violet staining was used to calculate lesion volumes, as described previously (see Supplemental materials for more details) [11].

### Immunohistochemistry

Immunohistochemistry was carried out on free floating brains (see Supplemental materials for more detail).

### Microscopy and image analysis

High power field images were collected on an Olympus BX51 upright microscope using a ×20 objective and captured using Coolsnap ES camera (photometrics, USA) through MetaVue software (Molecular Devices, USA). Images were then analysed with ImageJ software (National Institutes of Health, USA). For each animal, immunohistochemistry micrographs were analysed on coronal sections of the same co-ordinates (approximately bregma level 0.84 mm, according to mouse brain atlas, www.mouse.brain-map.org), which represented the middle of the infarcted territory from anterior-posterior perspective. Both ipsilateral and contralateral hemispheres in penumbra and core regions were measured (see Supplemental Fig. 1 for diagram). The % area of the total image stained with PECAM-1, VEGFR2, lectin, col IV, laminin, integrin-β1, and GFAP were quantified with ImageJ. In addition, VEGFR2 was normalised to PECAM-1, and col IV, laminin, and integrin-β1 were normalised to lectin (e.g. (% area staining col IV / % area staining lectin) × 100). Vessel diameter (μM) of col IV labelled vessels, the number of vascular GFAP^+^ astrocytes, peri-vascular GFAP^+^ astrocytes, vessels, KI-67^+^ vessels, and NeuN^+^ neurons were counted manually with ImageJ. For vessel diameter analysis, the vessel diameter of all vessels present in an image were analysed, and an overall mean vessel diameter was then calculated. The width of the centre of the vessel in μM was measured using ImageJ software. In instances where vessels were composed of multiple branches, the width of the centre of each branch was measured and an average value was calculated. Four images, i.e. 1 per region, per mouse were analysed. Number of KI-67^+^ vessels was also expressed as a % of total number of vessels. Iba1^+^ microglia were counted based on their activation status determined by their morphology as described previously [12]. All collection and analyses of images were carried out by a blinded experimenter.

### Statistical analyses

All data are presented as mean values ± standard deviation of the mean (SD). For LSCI analyses, linear mixed modelling was used to evaluate the effect of independent factors (genotype and time) on the dependent variable [13]. All factors and interactions were modelled as fixed effects. A within-subject design with random intercepts was used for all models. The significance of inclusion of a dependent variable or interaction terms was evaluated using log-likelihood ratio. Holm-Sidak post hocs were then performed for planned pair-wise comparisons using the least square means [14]. Homoscedasticity and normality were evaluated graphically using predicted vs residual and Q-Q plots, respectively. All analyses were performed using R (version 3.3.3). For the remaining data, normality was assessed with the Shapiro-Wilk test, and appropriate transformations were employed when necessary. Statistical analyses performed on normally distributed data were unpaired Student’s *t* test and repeated measures two-way analysis of variance (ANOVA) test with Sidak corrected post hoc test. The Mann-Whitney test was used for data that were not normally distributed. Accepted levels of significance were *P < 0.05, **P < 0.01, and ***P < 0.001. Statistical analyses were carried out using GraphPad Prism 7.0.

## Results

### PTX3 KO mice exhibit impaired long-term recovery of cerebral blood flow after ischaemic stroke

**Fig. 1:**
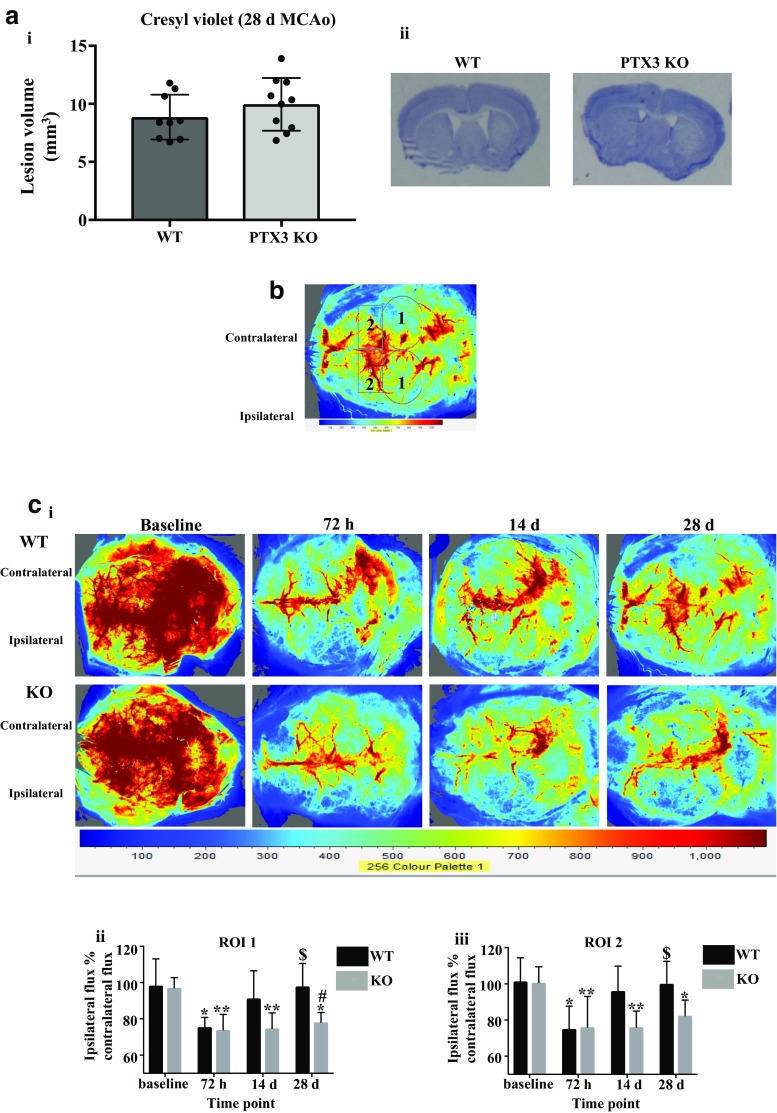
PTX3 promotes long-term CBF recovery after cerebral ischaemia. **a** Infarct volume 28 days after cerebral ischaemia of WT and PTX3 KO mice was assessed on cresyl violet stained brain sections. **b** Two regions of interest (ROIs) were used to quantify cerebral blood flow (CBF) in WT and PTX3 KO mice as follows; ROI 1 was primary middle cerebral artery (MCA) area and ROI 2 was frontal cortical region. Labels 1 correspond to ROI 1, labels 2 correspond to ROI 2. **c i** LSCI images at baseline, and 72 h, 14 days, and 28 days post-stroke in WT and PTX3 KO mice. **c ii**–**iii** CBF quantified with moorFLPI2 Full-Field Laser Perfusion Imager Review V5.0 software and expressed as ipsilateral flux as % of contralateral flux in two ROIs. Statistical analyses were performed using **a** unpaired Student’s *t* test (ns *P* > 0.05) and **c** linear mixed modelling followed by Holm-Sidak post hoc (ns *P* > 0.05, * *P* ≤ 0.05, ** *P* ≤ 0.01, *** *P* ≤ 0.001). * represents significance within genotype from baseline; $ represents significance within genotype from 72 h; # represents significance between genotype. All data are presented as mean ± SD (*n* = 6–7 per group)

Assessment of infarct volume using cresyl violet staining revealed no significant difference in lesion volumes in PTX3 KO mice compared to WT mice 28 days after MCAo (Fig. 1a). No significant differences in plasma levels of PTX3 were observed between WT and PTX3 KO mice 48 h after MCAo. In addition, there was no difference in infarct volume between the two genotypes 48 h after MCAo (Supplementary Fig. 4b). We have previously demonstrated histologically that PTX3 KO mice present impaired angiogenesis 14 days after cerebral ischaemia [5]. We therefore aimed to determine whether PTX3 KO mice have impaired recovery of CBF long-term (14 and 28 days) after ischaemic stroke (Fig. 1c). LSCI revealed a significant main effect of time (ROI 1 *P* < 0.001, ROI 2 *P* < 0.001), genotype (ROI 1 *P* < 0.01, ROI 2 *P* < 0.01), and interaction (ROI 1 *P* < 0.05, ROI 2 *P* < 0.05). In ROI 1 and ROI 2, there was no significant difference in baseline CBF (ipsilateral flux % contralateral flux) or CBF at 72 h, between WT and PTX3 KO mice. Both genotypes exhibited a significant (ROI 1 WT 23% and KO 24%; ROI 2 WT 26% and KO 24%) reduction in CBF at 72 h compared to baseline after cerebral ischaemia. We observed no recovery of CBF in PTX3 KO mice as they exhibited a significant reduction in CBF at 14 (ROI 1 23%, and ROI 2 24%) and 28 days (ROI 1 20% and ROI 2 18%) compared to baseline, whereas CBF in WT mice recovered with CBF levels returning to baseline levels. Furthermore, we found CBF in WT mice was significantly (ROI 1 30% and ROI 2 33%) increased at 28 days compared to 72 h. At 14 days, CBF was markedly (ROI 1 *P* = 0.08, ROI 2 *P* = 0.07) reduced in PTX3 KO mice compared to WT mice. At 28 days (ROI 1), PTX3 KO mice had significantly (20%) reduced CBF compared to WT mice, whilst in ROI 2 although not significant PTX3 KO mice demonstrated a marked (18%) reduction in CBF compared with WT mice (Fig. 1c). Collectively, these data suggest that long-term CBF recovery after cerebral ischaemia is impaired in PTX3 KO mice, suggesting a key role for PTX3 in long-term restoration of blood flow post-stroke.

### Lack of PTX3 impairs vascular remodelling after cerebral ischaemia

**Fig. 2:**
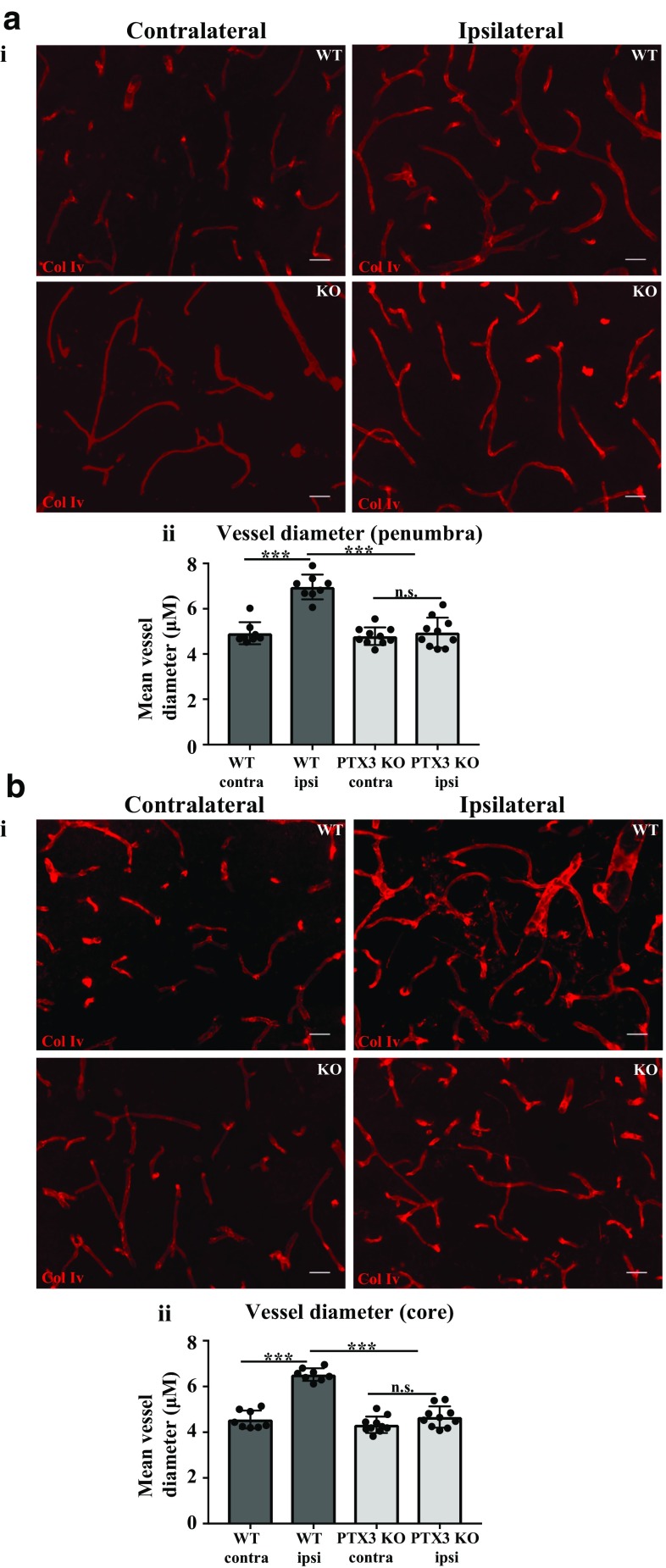
PTX3 KO mice have reduced vessel diameter 28 days after MCAo. **a i**, **b i** collagen IV (col IV)-labelled blood vessels (red) in ipsilateral and contralateral hemispheres of penumbra and core regions 28 days after MCAo as labelled. Scale bar 30 μM. **a ii**, **b ii** Mean vessel diameter (μM) in ipsilateral and contralateral hemispheres of penumbra and core regions of wild type (WT) and PTX3 knockout (KO) mice was assessed with ImageJ software. **a**–**b** Statistical analyses were performed using repeated measures two-way ANOVA followed by Sidak corrected post hoc analysis (ns *P* > 0.05, *** *P* ≤ 0.001). All data are presented as mean ± SD (*n* = 8–10)

Impaired CBF in PTX3 KO mice observed in our study led us to hypothesise that PTX3 regulates vessel diameter after experimental stroke. Previous studies have reported an increase in vessel diameter leads to an increase in CBF, 14 and 21 days after stroke [15]. Therefore, we assessed the morphology of vessels, measuring vessel diameter 28 days after induction of experimental stroke. Vessel diameter was significantly increased after stroke both in the penumbra (42%) and core (28%) region in WT mice, but not in PTX3 KO mice (Fig. 2a, b). In addition, PTX3 KO mice display significantly reduced vessel diameter in the ipsilateral hemispheres compared to WT mice, both in the penumbra and core region. Importantly, these data suggest that PTX3 may regulate vessel diameter.

### PTX3 is important for driving vessel-associated cellular proliferation 14 and 28 days post-stroke

**Fig. 3:**
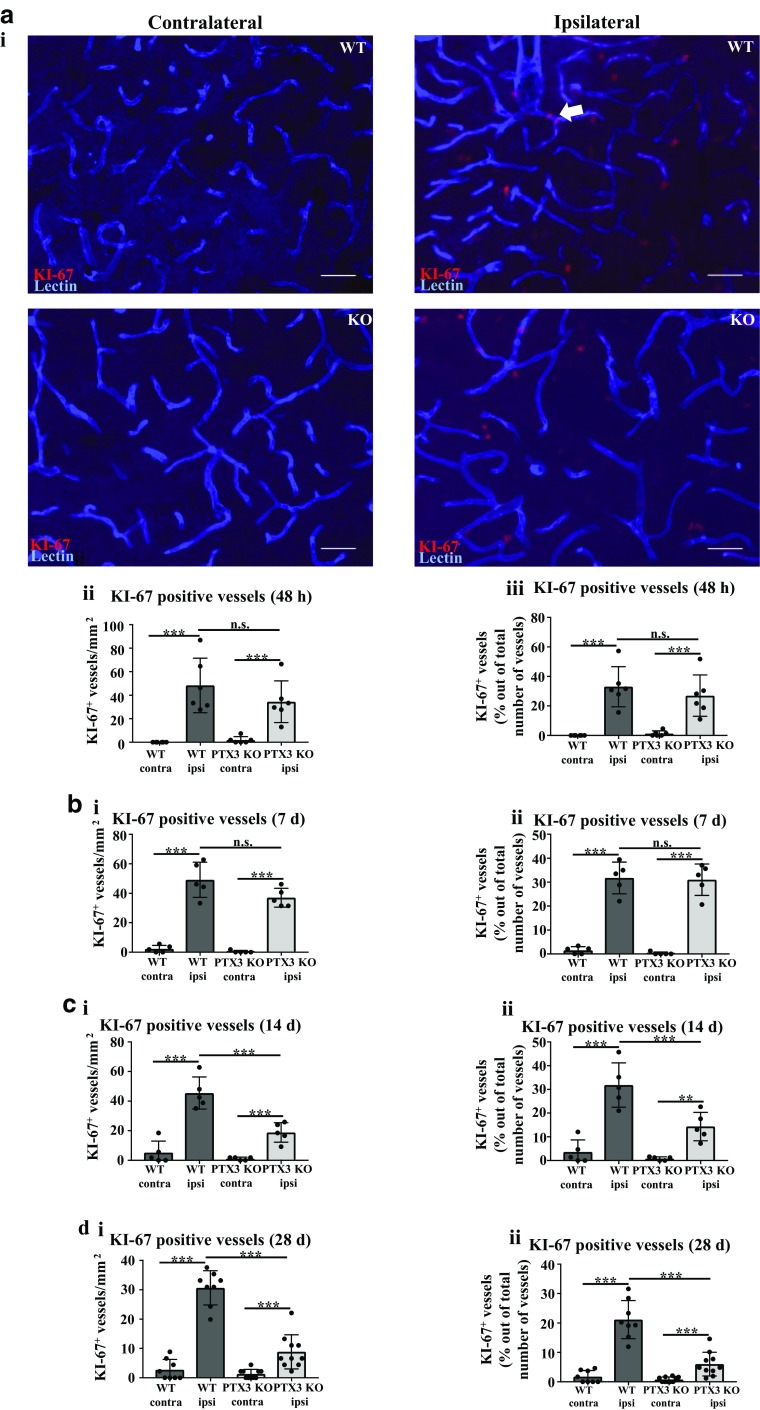
Lack of PTX3 impairs vessel proliferation 14 and 28 days after MCAo. **a i** KI-67 (red) and lectin (blue) co-immunohistochemistry of ipsilateral or contralateral hemispheres of penumbra region in wild type (WT) or pentraxin 3 knockout (PTX3 KO) mice. Scale bar 50 μM. **a ii**, **b i**, **c i**, **d i** Number of Ki-67-positive (+) blood vessels in the ipsilateral or contralateral hemisphere of penumbra region of WT or PTX3 KO mice was evaluated using Image J software. **a iii**, **b ii**, **c ii**, **d ii** quantification of number of KI-67^+^ vessels as a percentage (%) of total number of vessels present using ImageJ software. **a–d** Statistical analyses determined by repeated measures two-way ANOVA followed by Sidak corrected post-hoc analysis (ns *P* > 0.05, ** *P* ≤ 0.01, *** *P* ≤ 0.001). All data expressed as mean ± SD (*n* = 5–10)

Long-term recovery of CBF post stroke can be facilitated by angiogenesis, and endothelial cell proliferation is a critical step for angiogenesis and formation of blood vessels [16]. In order to examine the potential pro-angiogenic effect of PTX3, we investigated the effect of PTX3 deficiency on vessel proliferation. WT and PTX3 KO mice have significantly increased vessel proliferation 48 h, 7 days, 14 days, and 28 days after experimental stroke, indicated by a significantly higher number of KI-67^+^ vessels and number of KI-67^+^ vessels as a % of total number of vessels present (Fig. 3a–d). However, lack of PTX3 significantly reduced long-term vessel-associated cellular proliferation compared to WT mice in the ipsilateral hemisphere (14 and 28 days post-stroke) (Fig. 3c, d). Collectively, these findings suggest that PTX3 may enhance long-term post-stroke angiogenesis.

### PTX3 KO mice display impaired angiogenesis 28 days after experimental stroke

**Fig. 4:**
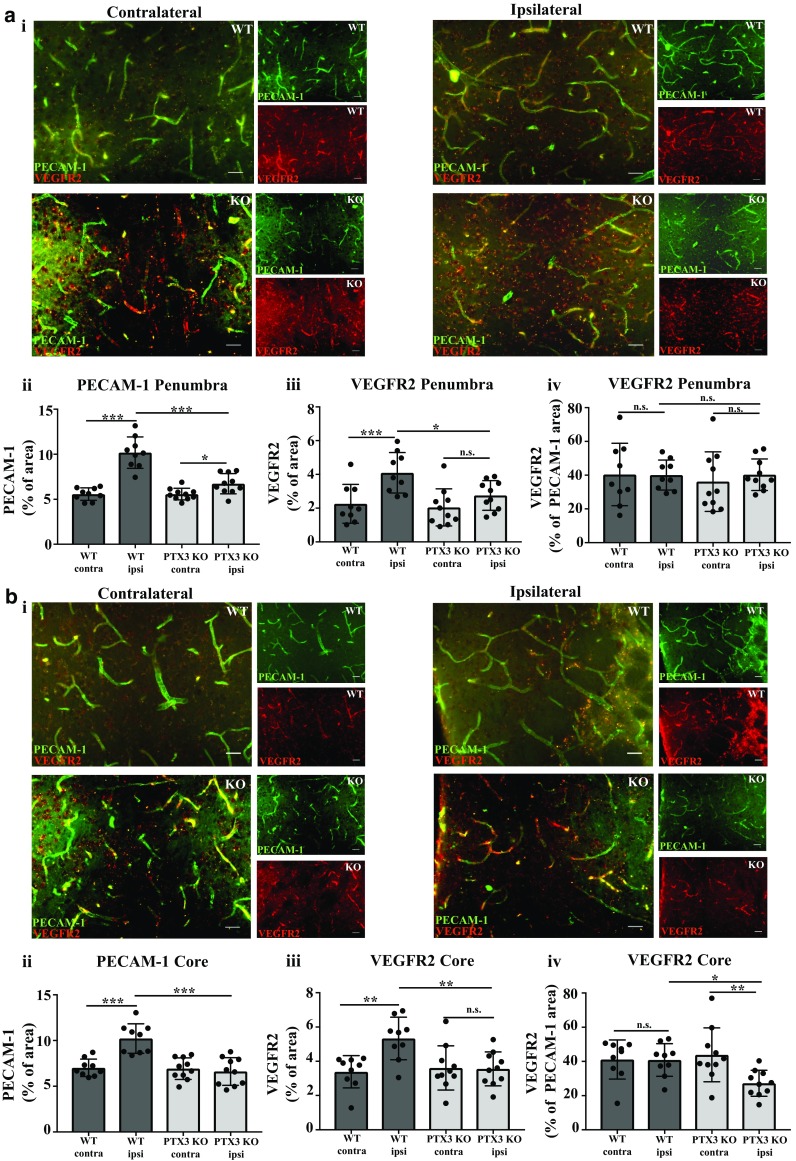
PTX3 KO mice have impaired angiogenesis 28 days after MCAo. **a i**, **b i** PECAM-1 and VEGFR2 co-immunohistochemistry, PECAM-1 (green), and VEGFR2 (red), 28 days after MCAo of ipsilateral (stroke) or contralateral hemispheres of penumbra and core regions as labelled. Scale bar 50 μM. Percentage (%) area of **a ii**, **b ii** PECAM-1, **a iii**, **b iii** VEGFR2, and **a iv**, **b iv** VEGFR2 as % of PECAM-1% area staining of ipsilateral and contralateral hemispheres of penumbra and core regions were quantified with ImageJ software. **a**–**b** Statistical analyses determined by using repeated measures two-way ANOVA followed by Sidak corrected post hoc analysis (ns *P* > 0.05, * *P* ≤ 0.05, ** *P* ≤ 0.01, *** *P* ≤ 0.001). All data expressed as mean ± SD (*n* = 9–10)

In order to determine whether the impaired CBF we observed in PTX3 KO mice after cerebral ischaemia (Fig. 1) is a result of impaired angiogenesis, we performed a study analysing long-term angiogenesis histologically 28 days after MCAo. Co-immunohistochemistry revealed a significant increase in PECAM-1 staining in the ipsilateral hemisphere compared to contralateral hemisphere in the penumbra region, in WT (83%) and PTX3 KO (21%) mice (Fig. 4a). In WT mice, we also observed a significant (46%) increase in PECAM-1 staining in the ipsilateral hemisphere compared to contralateral hemisphere in the core region (Fig. 4b). We observed a significant decrease in PECAM-1 staining in the ipsilateral hemisphere of PTX3 KO mice compared to ipsilateral hemisphere of WT mice, in both the penumbra (34%) and core (35%) regions (Fig. 4a, b). In WT mice, but not in PTX3 KO mice, VEGFR2 staining was significantly increased in the ipsilateral hemisphere when compared to the contralateral hemisphere, both in the penumbra (82%) and core (57%) regions. PTX3 KO mice exhibited a significant (penumbra 33%, core 33%) reduction in ipsilateral VEGFR2 staining in the ipsilateral hemispheres compared to ipsilateral hemispheres of WT mice (Fig. 4a, b). Our data indicated that VEGFR2 staining normalised to PECAM-1 staining in the ipsilateral hemisphere was significantly (18%) decreased in PTX3 KO mice compared to WT mice in the core region, whilst no significant difference was found in the penumbra (Fig. 4a, b). These data combined indicate for the first time that PTX3 is a key driver of long-term angiogenesis and vascular remodelling 28 days after stroke. To exclude the possibility that the angiogenesis and CBF phenotype are not caused by disturbed vascular development, vascular staining in coronal sections from healthy WT and PTX3 KO mice was analysed via lectin immunohistochemistry to identify vasculature. No significant difference between cerebral vasculature was observed between WT and PTX3 KO mice, suggesting that they do not have disturbed vasculature (Supplementary Fig. 3).

### PTX3 upregulates integrin-β1 expression in the cerebrovasculature 28 days after MCAo

**Fig. 5:**
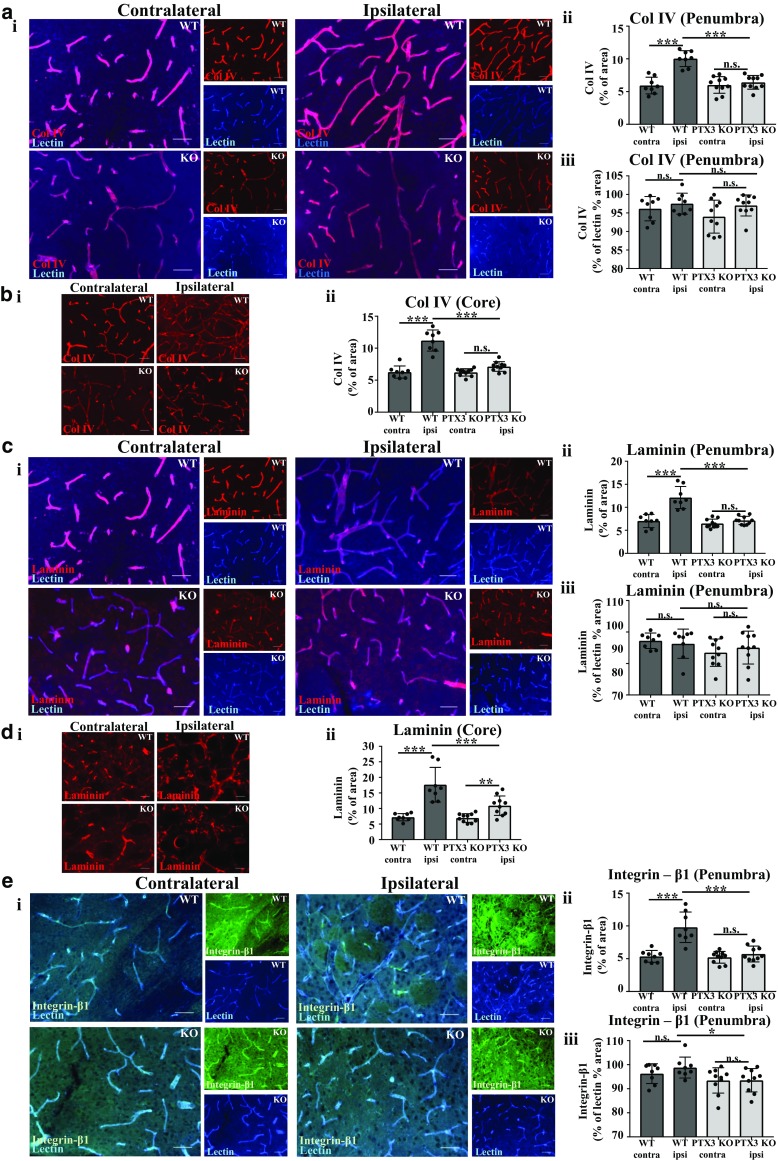
Vessel-based extracellular matrix (ECM) proteins collagen IV (col IV), laminin, and ECM receptor integrin-β1expression correlate with previously observed reduced vasculature in pentraxin 3 knockout (PTX3 KO) mice 28 days after MCAo. **a i**, **c i**, **e i** Vasculature labelled with lectin (blue) and co-immunostaining with col IV (red), laminin (red), or integrin-β1 (green), respectively, in ipsilateral and contralateral hemispheres of penumbra region as labelled. Scale bar 50 μM. **b i** col IV (red) and **d i** laminin (red) immunostaining of ipsilateral and contralateral hemispheres of the core region as labelled. Scale bar 50 μM. Percentage (%) area staining of col IV (**a ii**, **b ii**) laminin (**c ii**, **d ii**) or integrin-β1 (**e ii**) in ipsilateral and contralateral hemisphere was quantified using ImageJ software. % area staining of **a iii** col IV, **c iii** laminin, or **e iii** integrin-β1 as a % of lectin % area staining in the ipsilateral or contralateral hemisphere of the penumbra region was assessed with ImageJ software. Scale bar 50 μM. **a**–**e** Statistical analyses were assessed using repeated measures two-way ANOVA followed by Sidak corrected post hoc analysis (ns *P* > 0.05, * *P* ≤ 0.05, ** *P* ≤ 0.01, *** *P* ≤ 0.001). All data are presented as mean ± SD (*n* = 8–10)

Extracellular matrix (ECM) receptor integrin-β1 and ECM proteins (col IV and laminin) are key regulators of blood vessel formation and integrity [17]. Previous evidence showed that PTX3 can influence ECM remodelling [18]. To examine whether PTX3 influences vascular ECM proteins and receptors involved in blood vessel formation, immunohistochemistry was carried out for vascular col IV, laminin, and integrin-β1 co-stained with lectin to identify cerebral blood vessels (Fig. 5a–e). Our findings demonstrated that in WT mice, col IV, laminin, and integrin-β1 staining was significantly greater in the ipsilateral hemisphere compared to the contralateral hemisphere of the penumbra (col IV 69%, laminin 73%, and integrin-β1 83%) and also core (col IV 79% and laminin 145%, respectively) (Fig. 5a–e). PTX3 KO mice only had a significant increase in staining of laminin in their core (57%) ipsilateral hemisphere compared to contralateral hemisphere (Fig. 5d). Interestingly, PTX3 KO mice had significantly reduced ipsilateral staining of col IV (36%) and laminin (41%) in the penumbra and core (col IV 36%, laminin 38%) and integrin-β1 in the penumbra (42%), compared to WT mice ipsilateral hemisphere (Fig. 5a–e). Although no significant differences were observed in staining of col IV or laminin normalised to lectin staining, we found that staining of integrin-β1 normalised to lectin staining was significantly (5%) reduced in PTX3 KO mice compared to WT mice, in the ipsilateral hemisphere of the penumbra (Fig. 5a, c, e).

### Post-stroke expression of reactive GFAP^+^ astrocytes is promoted by PTX3

**Fig. 6:**
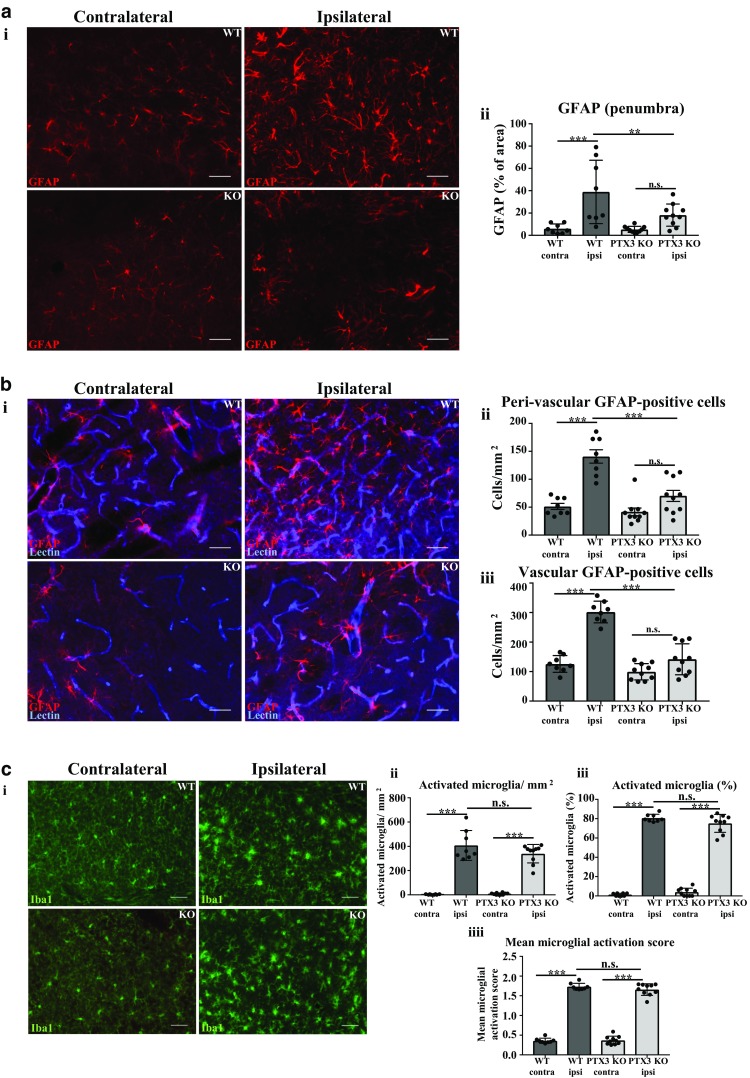
Pentraxin 3 knockout (PTX3 KO) mice express reduced number of vascular and peri-vascular GFAP-positive astrocytes. **a i** Glial fibrillary acidic protein (GFAP)-positive astrocytes (red) in ipsilateral or contralateral hemispheres of penumbra region in WT or PTX3 KO mice as labelled. Scale bar 50 μM. **b i** GFAP (red) and lectin (blue) co-immunohistochemistry of ipsilateral or contralateral hemispheres of penumbra region in WT or PTX3 KO mice as labelled. Scale bar 50 μM. **c i** Iba1 (green) expressing microglia in ipsilateral or contralateral hemispheres of penumbra region in WT or PTX3 KO mice as labelled. Scale bar 50 μM. **a ii** Quantification of GFAP percentage (%) area staining in ipsilateral or contralateral hemispheres of penumbra region in WT or PTX3 KO mice. **b ii** Peri-vascular or **b iii** vascular GFAP^+^ astrocytes/mm^2^ in ipsilateral or contralateral hemispheres of penumbra region in WT or PTX3 KO mice were counted with ImageJ software. The **c ii** number/mm^2^ or **c iii** % of Iba1 expressing microglia was evaluated with ImageJ software. **c iiii** Mean microglial activation score of Iba1 stained microglia was also calculated. Statistical analyses performed using repeated measures two-way ANOVA followed by Sidak corrected post hoc analysis (ns *P* > 0.05, ** *P* ≤ 0.01, *** *P* ≤ 0.001). All data expressed as mean ± SD (*n* = 8–10)

Studies have shown that astrocytes can promote angiogenesis via VEGF and Notch 1 signalling [19, 20] and neurovascular remodelling and recovery after stroke [21, 22], whereas microglia can produce angiogenic factors and promote angiogenesis after stroke [23–25]. Therefore, we investigated whether PTX3 affects GFAP^+^ astrocytes and/or Iba1^+^ microglia 28 days after experimental stroke. Immunohistochemistry quantification of GFAP staining revealed a significant (7-fold) increase in ipsilateral hemisphere compared to contralateral hemisphere in WT mice, but not in PTX3 KO mice (Fig. 6a). A significant (54%) reduction in GFAP staining was observed in the ipsilateral hemisphere of PTX3 KO mice compared to the ipsilateral hemisphere of WT mice.

The number of both peri-vascular (1.74-fold) and vascular (1.40-fold) GFAP^+^ astrocytes was found to be significantly greater in the ipsilateral hemisphere compared to the contralateral hemisphere in WT mice, but not in PTX3 KO mice (Fig. 6b). Moreover, PTX3 KO mice exhibited a significantly smaller number of both peri-vascular (50%) and vascular (53%) GFAP^+^ astrocytes in their ipsilateral hemisphere, when compared to WT ipsilateral hemisphere. In contrast, although both WT and PTX3 KO mice demonstrated significantly increased number % and mean activation score of activated microglia, in their ipsilateral hemisphere compared to contralateral hemisphere, our data indicated no significant difference between genotypes (Fig. 6c). Consequently, these results demonstrate for the first time another potential post-stroke pro-angiogenic mechanism driven by PTX3 involving reactive astrocytes.

### PTX3 reduces neuronal loss 28 days after experimental stroke

**Fig. 7:**
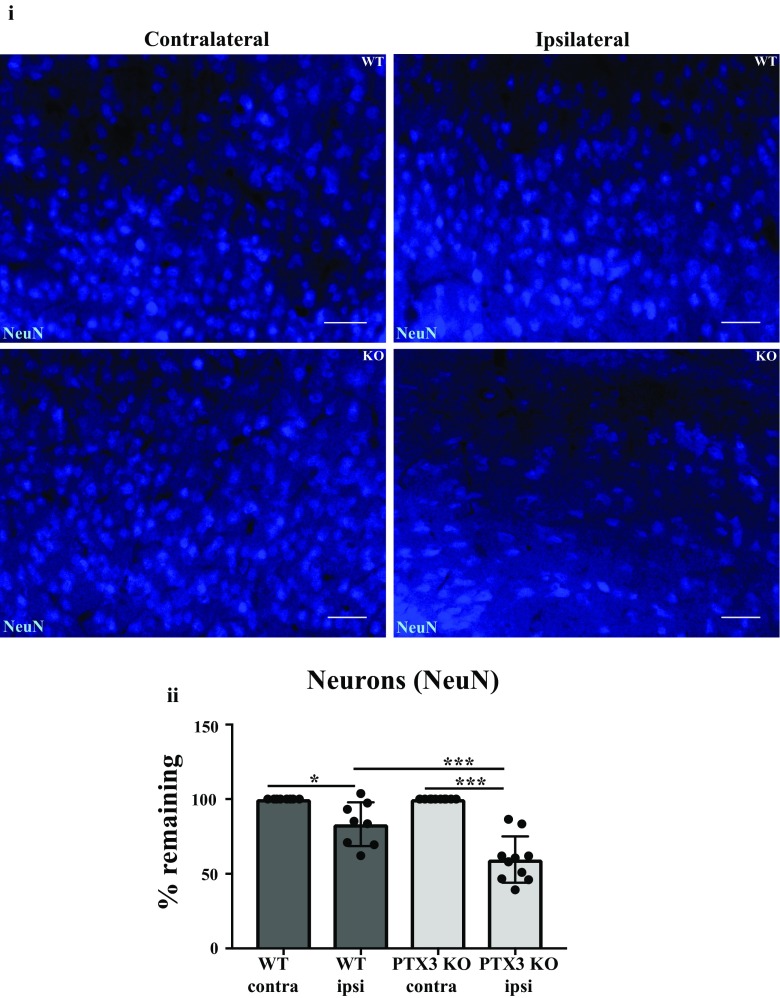
Pentraxin 3 (PTX3) is neuroprotective 28 days after MCAo. **i** Immunohistochemistry of NeuN (blue) positive (+) neurons in ipsilateral or contralateral hemispheres of penumbra region in wild type (WT) or PTX3 knockout (KO) mice as labelled. Scale bar 50 μM. **ii** Number of NeuN^+^ neurons in the ipsilateral and contralateral hemispheres of the penumbra region of WT and PTX3 KO mice were counted using ImageJ software. The number of NeuN^+^ neurons present in the ipsilateral hemisphere was calculated as a percentage of NeuN^+^ neurons counted in the corresponding contralateral hemisphere for WT and PTX3 KO mice. Statistical analyses assessed using repeated measures two-way ANOVA followed by Sidak corrected post hoc analysis (* *P* ≤ 0.05, *** *P* ≤ 0.001). All data expressed as mean ± SD (*n* = 8–10)

Our research group has previously demonstrated that PTX3 can promote neurogenesis after experimental ischaemic stroke [5]. Additionally, there is emerging evidence indicating that reactive astrocytes are neuroprotective in ischaemic stroke [26]. Considering these previous findings and our data in this study suggesting that PTX3 promotes expression of GFAP^+^ astrocytes, we assessed whether PTX3 influences neuronal viability after experimental stroke. We found a significant reduction in % of remaining neurons in the ipsilateral hemisphere compared to the contralateral hemisphere, in both WT (17%) and PTX3 KO (40%) mice. Importantly, PTX3 KO mice exhibited a significant (28%) reduction in % of neurons remaining in their ipsilateral hemisphere compared to ipsilateral hemisphere of WT mice (Fig. 7). These findings suggest that PTX3 reduces neuronal loss after stroke. We did not observe any significant differences in behaviour assessed by 28-point neuroscore test, foot fault test and open field test (Supplementary Fig. 5).

## Discussion

To our knowledge, the role of the acute phase protein PTX3 on CBF after cerebral ischaemia has not been previously studied, and here, we are the first to show that PTX3 is a key mediator of long-term CBF recovery. Specifically, we demonstrated that in the primary MCA area and frontal cortical region, PTX3 KO mice showed no recovery in CBF. In contrast, mice expressing PTX3 showed a marked increase in CBF at 14 days and significantly increased CBF 28 days after stroke, when compared to CBF levels at 72 h. Importantly, in WT mice, CBF was close to baseline levels at 14 days and returned to baseline values by 28 days. In addition, vascular remodelling (i.e. increased vessel diameter) occurs in the ischaemic hemisphere of WT mice, but not PTX3 KO mice, after cerebral ischaemia. These observations are consistent with a previous report showing that vessel diameter increases alongside recovery of CBF during the sub-acute phase after cerebral ischaemia [15]. Therefore, we believe that the increased vessel diameter and/or neoangiogenesis observed in WT mice in our study may have contributed to the increased CBF we observed in these mice. It is possible that these neosynthesised vessels are perfused; however, this requires further experimental investigation. Collectively, our findings are clinically important as they strongly suggest that PTX3 aids long-term recovery of CBF after cerebral ischaemia, possibly mediated by mechanisms of ECM remodelling and angiogenesis, and therefore that upregulating PTX3 is a promising therapeutic strategy to improve outcome in stroke patients.

There is robust evidence demonstrating that endothelial cell proliferation, which is a key angiogenic response, directly leads to an increase in vessel diameter [27, 28]. Our study further demonstrated that lack of PTX3 significantly impaired long-term angiogenesis after cerebral ischaemia. Indeed, vessel-associated cell proliferation was markedly decreased in PTX3 KO mice compared to WT mice, and vessel density and vascular VEGFR2 expression were also significantly decreased in PTX3 KO mice compared to WT mice, up to 28 days after MCAo. These data build on our previous finding that PTX3 KO mice have impaired angiogenesis 14 days after cerebral ischaemia [5], but show for the first time that long-term (28 days) post-stroke angiogenesis regulated by PTX3 correlates with total recovery of CBF. A previous study has reported that an acidic microenvironment, similar to acidosis pH levels observed in ischaemic stroke, activates PTX3 into a tissue remodelling and repair mode in a lung injury model [29]. The remodelling of the ECM has been described previously as an important factor regulating vessel diameter and angiogenesis [28]. In particular, integrin-β1 is critically important for vessel formation and integrity, and studies blocking integrin-β1 have demonstrated a significant reduction in vessel diameter [17, 30]. Our study also found that expression of vessel-associated integrin-β1, as well as ECM proteins col IV and laminin, and perivascular astrocytes were all reduced by the lack of PTX3, 28 days after cerebral ischaemia, further suggesting that cerebrovascular ECM remodelling regulated by PTX3 may be a key step for restoration of CBF after stroke. Interestingly, our study also found that PTX3 KO mice exhibit a significant decrease in neuronal integrity compared to WT mice 28 days after MCAo. A previous study already demonstrated the acute neuroprotective effect of PTX3 in a mouse model of seizure [31]. Lack of PTX3 had no effect on acute neurodegeneration in our study and as previously reported by us [6], but our data suggest that PTX3 may support neuronal survival during the sub-acute phase of stroke. Microglia are key contributors to neuronal survival in the injured brain and a recent report demonstrated protective action of microglia early after stroke [32]. We found that activated microglia are present in ischaemic hemispheres of both WT and PTX3 KO mice, although no significant difference was observed between genotypes, suggesting that PTX3 may support long-term neuronal survival independently of microglial activity. We did not observe any functional behavioural differences between WT and PTX3 KO mice. The discrepancy between structural and functional outcome in our study may suggest that better refined behavioural tests are needed in order to investigate the role of PTX3 on functional recovery.

In summary, we have shown that PTX3 is a key promoter of sustained long-term CBF recovery, angiogenesis, and neuronal viability 28 days after cerebral ischaemia, demonstrating its beneficial clinical potential. We propose that PTX3 can be targeted to promote long-term neurovascular repair and protect neurons after ischaemic stroke and possibly other cerebrovascular ischaemic injuries.

## Electronic supplementary material

ESM 1 (PPTX 30571 kb)

ESM 2 (DOCX 30 kb)
