# Supplementary material for: Pentraxin 3 promotes long-term cerebral blood flow recovery, angiogenesis, and neuronal survival after stroke
Source: J Mol Med (Berl). 2018 Oct 13;96(12):1319–32. doi: 10.1007/s00109-018-1698-6 (PMC6245246; doi:10.1007/s00109-018-1698-6)
Supplement: Supplementary file 1 — (PPTX 30571 kb) [file 109_2018_1698_MOESM1_ESM.pptx]

## Slide 1
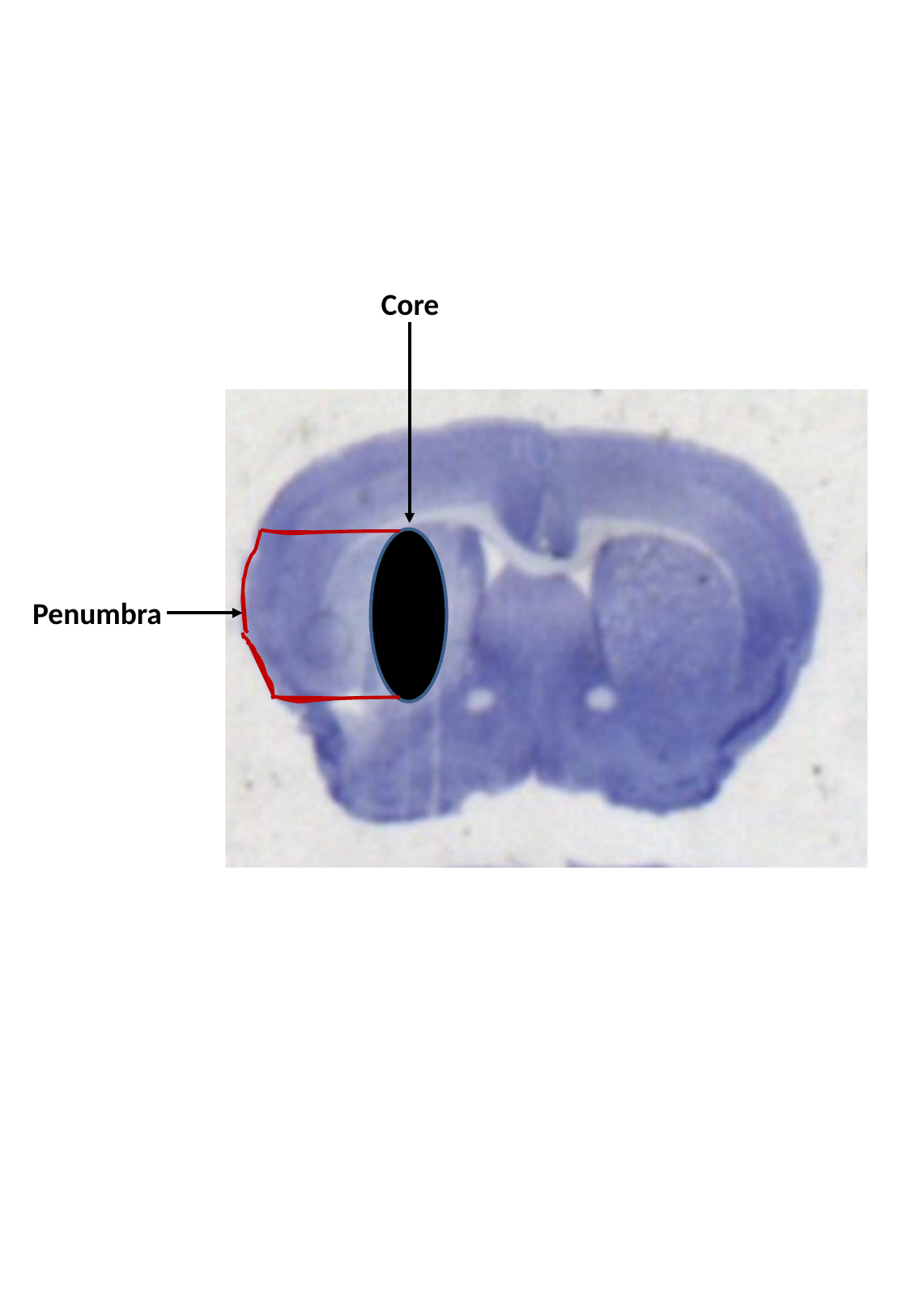

Core
Penumbra

## Slide 2
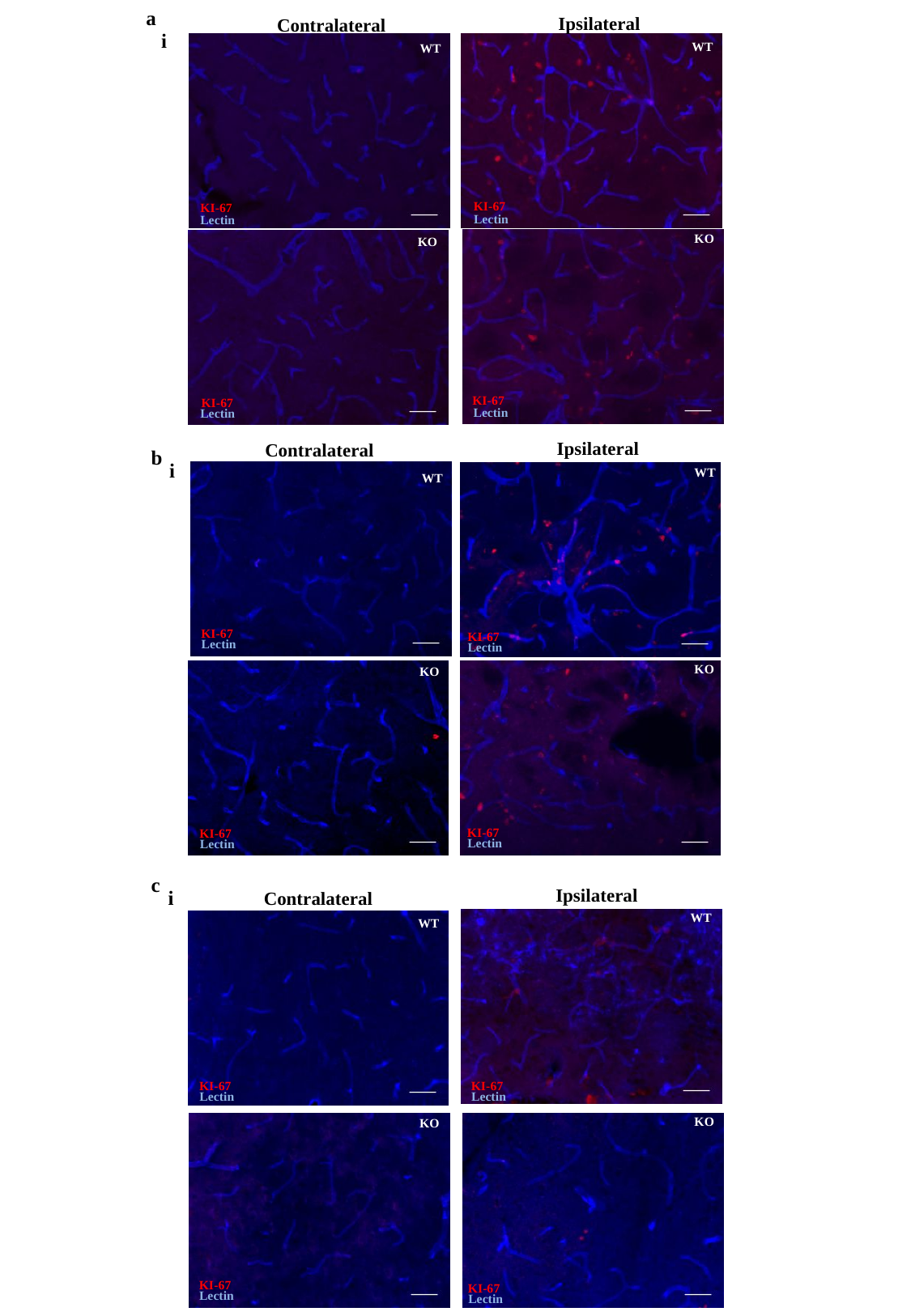

a
Ipsilateral
Contralateral
i
WT
WT
KI-67
KI-67
Lectin
Lectin
KO
KO
KI-67
KI-67
Lectin
Lectin
Ipsilateral
Contralateral
b
i
WT
WT
KI-67
KI-67
Lectin
Lectin
KO
KO
KI-67
KI-67
Lectin
Lectin
c
Ipsilateral
i
Contralateral
WT
WT
KI-67
KI-67
Lectin
Lectin
KO
KO
KI-67
KI-67
Lectin
Lectin

## Slide 3
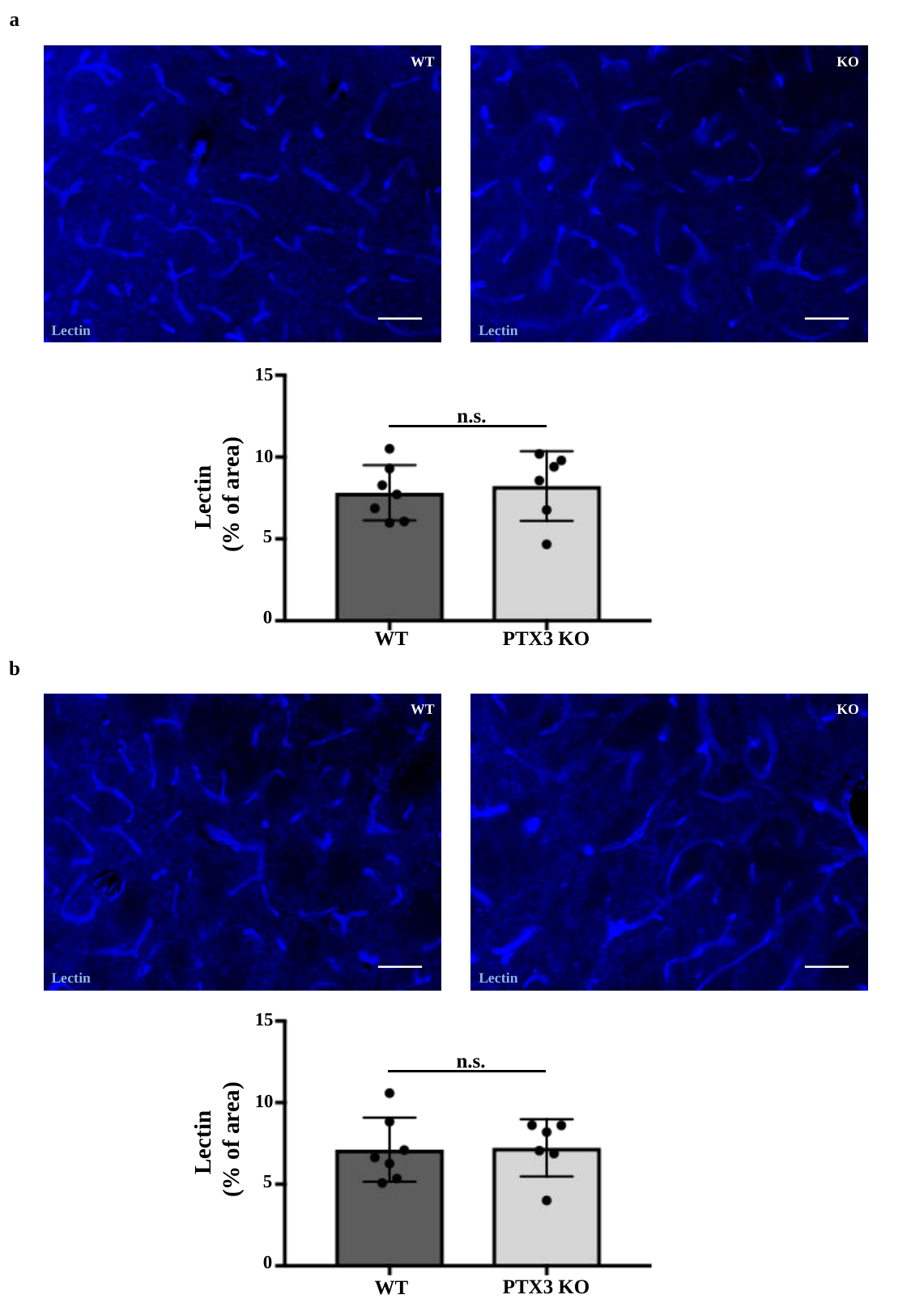

a
KO
WT
Lectin
Lectin
15
n.s.
10
Lectin
 (% of area)
5
0
PTX3 KO
WT
b
KO
WT
Lectin
Lectin
15
n.s.
10
Lectin
 (% of area)
5
0
PTX3 KO
WT

## Slide 4
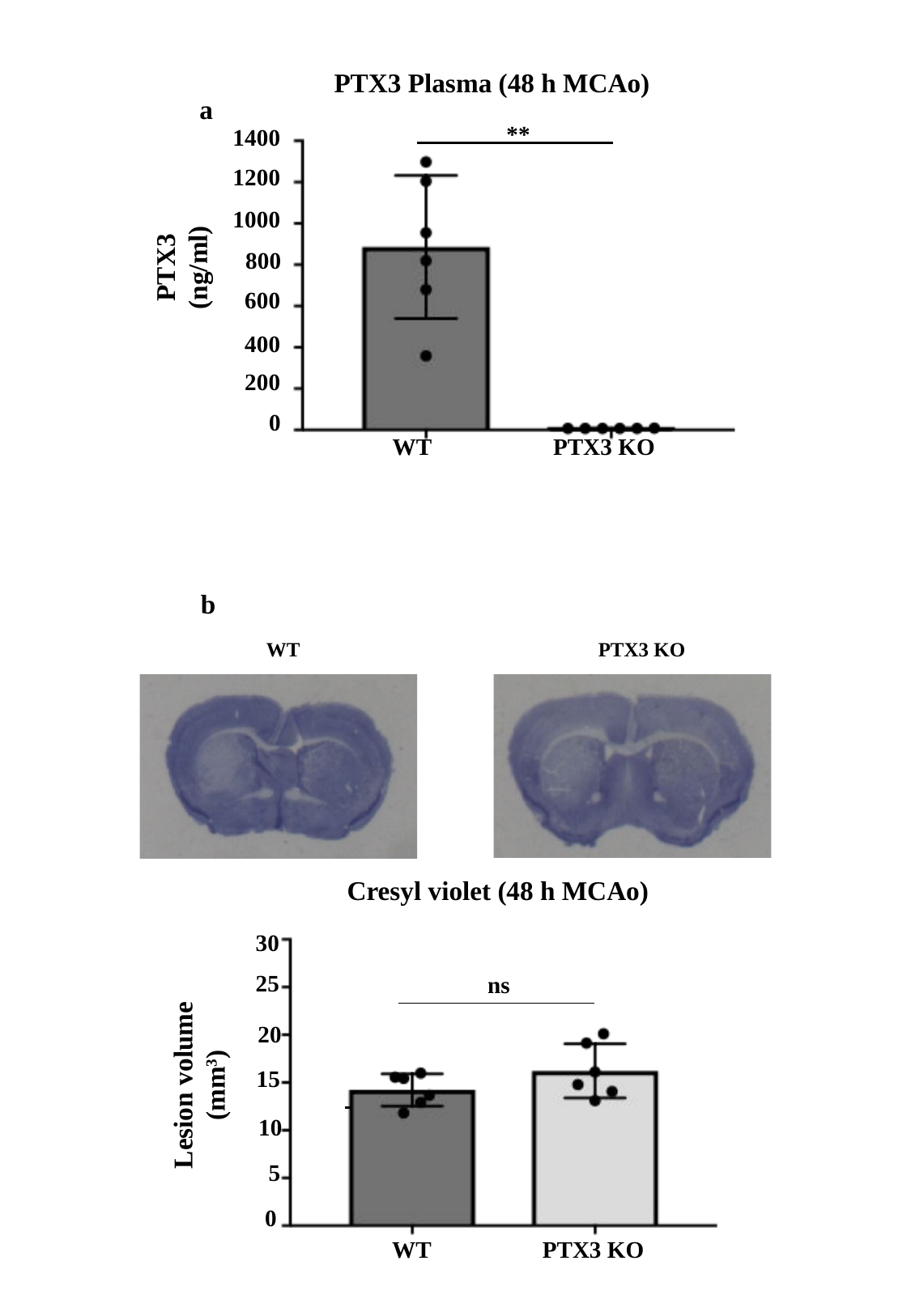

PTX3 Plasma (48 h MCAo)
a
**
1400
1200
1000
PTX3 (ng/ml)
800
600
400
200
0
PTX3 KO
WT
b
WT
PTX3 KO
Cresyl violet (48 h MCAo)
30
25
ns
20
Lesion volume (mm3)
15
n.s.
10
5
0
PTX3 KO
WT

## Slide 5
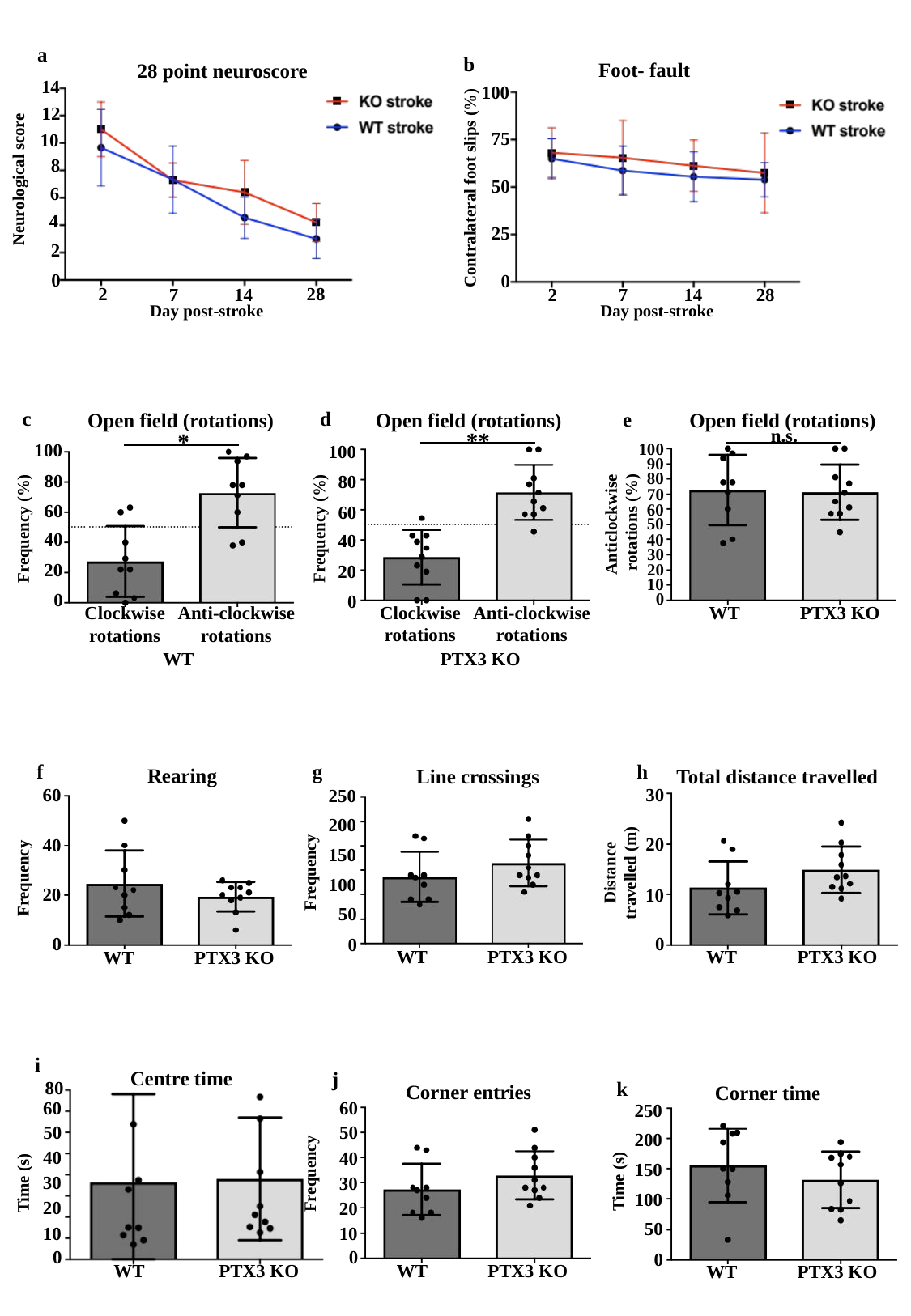

a
b
Foot- fault
28 point neuroscore
14
100
12
75
10
8
Neurological score
50
Contralateral foot slips (%)
6
4
25
2
0
0
28
2
7
14
28
2
7
14
Day post-stroke
Day post-stroke
c
d
e
Open field (rotations)
Open field (rotations)
Open field (rotations)
n.s.
**
*
100
100
100
90
80
80
80
70
60
60
60
Anticlockwise
rotations (%)
50
Frequency (%)
Frequency (%)
40
40
40
30
20
20
20
10
0
0
0
Clockwise rotations
Anti-clockwise rotations
WT
Clockwise rotations
Anti-clockwise rotations
PTX3 KO
PTX3 KO
WT
f
g
h
Rearing
Line crossings
Total distance travelled
60
30
250
200
20
40
150
Distance travelled (m)
Frequency
Frequency
100
20
10
50
0
0
0
WT
PTX3 KO
WT
PTX3 KO
WT
PTX3 KO
i
Centre time
j
k
80
Corner entries
Corner time
60
60
250
50
50
200
40
40
150
Frequency
30
Time (s)
30
Time (s)
100
20
20
50
10
10
0
0
0
WT
PTX3 KO
WT
PTX3 KO
WT
PTX3 KO
