# Supplementary material for: Pentraxin 3 promotes long-term cerebral blood flow recovery, angiogenesis, and neuronal survival after stroke
Source: J Mol Med (Berl). 2018 Oct 13;96(12):1319–32. doi: 10.1007/s00109-018-1698-6 (PMC6245246; doi:10.1007/s00109-018-1698-6)
Supplement: Supplementary file 2 — (DOCX 30 kb) [file 109_2018_1698_MOESM2_ESM.docx]

**Pentraxin 3 promotes long-term cerebral blood flow recovery, angiogenesis and neuronal survival after stroke**

Ivana Rajkovic^1^, Raymond Wong^1^, Eloise Lemarchand^1^, Jack Rivers-Auty^1^, Olivera Rajkovic^1^, Cecilia Garlanda^2^, Stuart M. Allan^1*^, and Emmanuel Pinteaux^1*^

**Supplemental Material**

**Supplemental Materials and Methods**

**Animals**

PTX3 KO mice and WT littermates were bred from heterozygote mice (obtained from Dr. Cecilia Garlanda, Humanitas Clinical and Research Center, Rozzano, Italy), and were genotyped as described previously (1). Experiments were conducted on weight-matched 12 – 14-week-old WT or PTX3 KO males. Animals were maintained under standard laboratory conditions: temperatures of 21 ± 1°C, 55 ± 10% humidity, 12-hour (h) light-dark cycle, ad libitum access to food and water. All animal procedures were carried out in accordance with the Animal Scientific Procedures Act (1986) and the European Council Directive 2010/63/EU, and were approved by the Home Office and Animal Welfare and Ethics Review Board, University of Manchester (UK). All experiments adhered to the ARRIVE guidelines and IMPROVE guidelines (2,3). Sample size for experimental stroke studies were determined by a priori power calculation using G*Power 3.1.9.2. Surgical procedures and behavioral tests were carried out by an experimenter blinded to the genotype.

**Behavioural testing**

All animals were assessed for neurological deficits 48 h post-MCAo, with animals recovering for 28 d also being assessed 7 d, 14 d and 28 d post MCAo, using a 28-point scoring system and a foot fault test as previously described (4,5). For the 28-point neuroscore test, animals were scored for seven behaviours with the sum of the scores used as the overall measure of the motor deficit for each individual mouse. A maximum score of 28 indicated the most severe level of neurological deficit (4). During the foot fault test, animals were positioned on to an elevated grid (30L × 30W × 31H cm) consisting of 2.5 cm^2^ sized squares and the number of foot faults (defined as the failure to step on the wire grid) of the ipsilateral and/or contralateral limbs were noted. Subsequently, % of contralateral foot faults was calculated (5). Exploratory behavior and motor function were also evaluated 3 d post MCAo using the open field test previously described (6). Briefly, animals were placed in the center of a square opaque Perspex box (45 cm x 30cm x 45 cm) for 5 min, which was recorded with a camera located above the box. ANY-maze tracking software (Stoelting Co.) was used to track the movement of mice, calculating parameters such as frequency of line crossings.

**Tissue processing**

Animals were anaesthetised with 4 % isoflurane and transcardially perfused with 0.9% saline followed by 4% paraformaldehyde (PFA) 48 h, 7 d, 14 d or 28 d after MCAo surgery. Brains were then dissected out and post-fixed in 4% PFA for 24 h, and subsequently transferred to 30% sucrose for a further 24 h. Brains were then frozen in isopentane (Fisher Scientific, UK) maintained at -30°C with dry ice, and stored at -80°C. Prior to perfusion, cardiac blood was extracted from the right ventricle, and then centrifuged at 2000 g, 4°C for 10 min, in order to isolate plasma from blood cells. For immunohistochemistry and Cresyl violet staining, brains were serially sectioned into 30 μm sections on a freezing sledge microtome (Bright, Cambridgeshire, UK). Sections were stored in cryoprotectant solution (30% ethylene glycol, 20% glycerol, 0.66 % sodium phosphate dibasic dehydrate, 0.079 % sodium dihydrogen orthophosphate 1-hydrate, in distilled water (dH_2_O)) at -20°C.

**Infarct volume analysis**

Cresyl violet staining was used to calculate lesion volumes, as described previously (7). In short, 8 coronal sections per brain spaced 360 µm apart were stained with cresyl violet, and infarcts were measured using Image J software. A total lesion volume per brain was calculated by combining the brain co-ordinates and lesion for each coronal section. Infarct size was corrected for oedema by calculating the increase in volume of the ipsilateral hemisphere compared to the contralateral hemisphere. All analyses were performed by a blinded experimenter.

**Enzyme linked immunosorbent assay (ELISA)**

Plasma samples from WT and PTX3 KO mice were assayed for PTX3 (pg/ml) by ELISA (R & D systems, UK), following the manufacturer’s instructions. Optical densities were read in a plate reader at 450 nm corrected for baseline at 570 nm (Synergy HT plate reader, BioTek). Concentrations of the samples were achieved by interpolating from a standard curve fitted by sigmoidal APL equation.

**Immunohistochemistry**

Free floating brain sections were washed three times with phosphate buffered saline (PBS). Antigen retrieval was carried out for col IV, laminin, and integrin-β1 antibodies prior to the blocking step as follows: free floating sections were transferred to 1.5 ml eppendorfs containing trisodium citrate (pH 8.5) diluted in dH_2_O, and incubated at 80°C for 30 min. All sections were then incubated with blocking buffer comprising of 10 % normal donkey serum (NDS) (Jackson laboratories, Bar Harbor, ME, USA), 0.3 % Triton X-100 (Sigma-Aldrich, UK) in PBS for 1 h. Blocking buffer was removed and sections were incubated with primary antibody diluted in primary antibody buffer consisting of 2% NDS, 0.3 % Triton X-100 and PBS, and left overnight at 4^o^C. Primary antibodies used in the study were as follows; rat anti-PECAM-1 (1:200, R& D systems, UK), goat anti-VEGFR2 (1:100, Abcam, UK), Lectin (1:100, Lycopersicon esculentum, Vector Laboratories Ltd, UK), rabbit anti-col IV (1:400, Abcam, UK), rabbit anti-laminin (1:100, Abcam, UK), rat anti-Integrin-β1 (1:100, Millipore, UK), chicken anti-GFAP (1:1000, Abcam, UK), rabbit anti-Iba1(1:1000, Wako, USA), mouse anti-NeuN (1:1000, Millipore, USA) and mouse anti-KI-67 (1:100, R& D systems, UK). Sections were washed three times with PBS and then incubated in the dark for 2 h with secondary antibodies diluted in primary antibody buffer. Secondary antibodies consisted of Alexa-Fluor 488 (1:500, rat, rabbit, ThermoFisher, UK), Alexa-Fluor 594 (1:500, goat, rabbit, chicken, mouse, ThermoFisher, UK) and Alexa-Fluor 350 (1:100, streptavidin, ThermoFisher, UK). Sections were then washed three times in PBS, mounted onto glass slides, and dried in the dark at RT for 24 h. Slides were then cover slipped with Prolong Gold with DAPI mounting agent (ThermoFisher, UK).

**Supplementary figure 1. Example of core and penumbra regions used for imaging and analysis of immunohistochemistry micrographs.** Core region is labelled as the area within the dark circle whereas penumbra region is labelled as the area inside of the red dotted lines. The core region was defined as the area of the lesion exposed to the most dramatic reduction in blood flow and greatest degree of damage (central striatal region). The penumbra was defined as the area receiving a smaller degree of reduction of blood flow and therefore less damage (salvageable tissue) i.e. the striatal and cortical area bordering the lateral core area.

**Supplementary figure 2. Vessel proliferation 7d, 14 d and 28 d after cerebral ischaemia.** (a i, b i and c i) Ki-67 (red) and lectin (blue) co-immunohistochemistry of ipsilateral or contralateral hemispheres of penumbra region in wild type (WT) or pentraxin 3 knock out (PTX3 KO) mice. Scale bar 50 μM.

**Supplementary figure 3. Vascular staining in naïve wild type (WT) and pentraxin 3 knock out (KO) mice.** (A and B) lectin (blue) immunohistochemistry staining of the vasculature in naïve WT and PTX3 KO mice in penumbral and core regions (corresponding to those defined in the 28 d MCAo study). Scale bar 50 μM. An unpaired student’s t-test (ns P > 0.05) revealed no significant difference in vascular staining (lectin % area) was observed between WT and PTX3 KO mice both in the penumbral and core region. All data are presented as mean ± SD (n = 6-7 per group).

**Supplementary figure 4. Pentraxin 3 (PTX3) plasma levels and lesion volume 48 h after MCAo in wild type (WT) and PTX3 knock out (KO) mice.** (A) PTX3 levels (pg/ml) in cardiac plasma collected 48 h after MCAo, with ELISA. In WT mice, plasma PTX3 levels were 885.6 pg/ml, whilst levels of PTX3 were below the limit of detection in PTX3 KO mice (8.127 pg/ml). (B) Lesion volumes assessed by cresyl violet staining in WT and PTX3 KO mice 48 h after MCAo. An unpaired student’s t-test (ns P > 0.05) revealed no significant difference in lesion volume between WT and PTX3 KO mice. All data are presented as mean ± SD (n = 6 per group).

**Supplementary figure 5. No significant differences in behaviour observed between wild type (WT) and pentraxin 3 knock out (PTX3 KO) mice after MCAo.** (A, B) Neurological deficits were assessed using the 28 point neuroscore and foot fault test (% of contralateral foot slips were counted), respectively. (C-K) Anxiety and exploratory behaviour was assessed using the open field test and analysed with ANY-maze software. (C, D, E) Frequency of clockwise and anticlockwise rotations in WT or PTX3 KO mice, and % of anticlockwise rotations WT vs PTK3 KO was determined. (F, G, J) Frequency of rearing, line crossings and corner entries, respectively. (H) Total distance travelled (metres, m). (I, K) Time spent in centre or corner squares (seconds, s), respectively. Statistical analyses determined using (B) repeated measures two-way ANOVA followed by Sidak corrected post-hoc analysis (ns P > 0.05), (A) Mann-Whitney test performed per day (ns P > 0.05), (C, D) one-sample t-test versus hypothetical value of 50 % (* P ≤ 0.05, ** P ≤ 0.01), (E-K) unpaired student’s t-test (ns P > 0.05). All data expressed as mean ± SD (n = 9-10).

**References**

1. Garlanda C, Hirsch E, Bozza S, Salustri A, De Acetis M, Nota R, et al. Non-redundant role of the long pentraxin PTX3 in anti-fungal innate immune response. Nature [Internet]. 2002;420(6912):182–6. Available from: http://www.nature.com/doifinder/10.1038/nature01195

2. Kilkenny C, Browne WJ, Cuthill IC, Emerson M, Altman DG. Improving bioscience research reporting: The arrive guidelines for reporting animal research. Animals. 2013;4(1):35–44.

3. Percie du Sert N, Alfieri A, Allan SM, Carswell HV, Deuchar GA, Farr TD, et al. The IMPROVE Guidelines (Ischaemia Models: Procedural Refinements Of in Vivo Experiments). J Cereb Blood Flow Metab. 2017 Nov;37(11):3488–517.

4. Clark W, Gunion-Rinker L, Lessov N, Hazel K. Citicoline treatment for experimental intracerebral hemorrhage in mice. Stroke. 1998;29(10):2136–40.

5. Balkaya M, Kröber JM, Rex A, Endres M. Assessing post-stroke behavior in mouse models of focal ischemia. J Cereb Blood Flow Metab [Internet]. 2013 Mar 12;33(3):330–8. Available from: http://www.ncbi.nlm.nih.gov/pmc/articles/PMC3587814/

6. Hall CS. Emotional behavior in the rat. I. Defecation and urination as measures of individual differences in emotionality. J Comp Psychol. 1934;18(3):385–403.

7. McColl BW, Rothwell NJ, Allan SM. Systemic Inflammatory Stimulus Potentiates the Acute Phase and CXC Chemokine Responses to Experimental Stroke and Exacerbates Brain Damage via Interleukin-1- and Neutrophil-Dependent Mechanisms. J Neurosci [Internet]. 2007;27(16):4403–12. Available from: http://www.jneurosci.org/cgi/doi/10.1523/JNEUROSCI.5376-06.2007
